# Effects of spontaneous first breath on placental transfusion in term neonates born by cesarean section: A randomized controlled trial

**DOI:** 10.3389/fped.2022.925656

**Published:** 2022-09-13

**Authors:** Hasan Kilicdag, Deniz Parlakgumus, Suleyman Cansun Demir, Mehmet Satar

**Affiliations:** ^1^Division of Neonatology, Department of Pediatrics, Acibadem Adana Hospital, Adana, Turkey; ^2^Department of Pediatrics, Acibadem Adana Hospital, Adana, Turkey; ^3^Division of Maternal Fetal Medicine, Department of Obstetrics and Gynecology, Cukurova University, Adana, Turkey; ^4^Division of Neonatology, Department of Pediatrics, Cukurova University, Adana, Turkey

**Keywords:** placental transfusion, physiologic-based cord clamping, umbilical cord milking, delayed cord clamping, neonates, infants

## Abstract

**Background:**

The role of umbilical cord management in placental transfusion in cesarean section (CS) requires clarification. The spontaneous first breath may be more important than the timing of cord clamping for placental transfusion in neonates born by CS.

**Objective:**

This study aimed to evaluate the impact of cord clamping after the first spontaneous breath on placental transfusion in neonates born by CS.

**Methods:**

We recruited women with a live singleton pregnancy at ≥37.0 weeks of gestation admitted for CS. The interventions performed, such as physiologic-based cord clamping (PBCC), intact-umbilical cord milking (I-UCM), 30-s delay in cord clamping (30-s DCC), and 60-s delay in cord clamping (60-s DCC), were noted and placed in a sealed envelope. The sealed envelope was opened immediately before delivery to perform randomization.

**Results:**

A total of 123 infants were eligible for evaluation. Of these, 31, 30, 32, and 30 were assigned to the PBCC, I-UCM, 30-s DCC, and 60-s DCC groups, respectively. The mean hemoglobin (Hb) and mean hematocrit (Hct) were significantly higher in the 60-s DCC group than in the PBCC group (*p* = 0.028 and 0.019, respectively), but no difference was noted among the I-UCM, 30-s DCC, and PBCC groups at 36 h of age. Further, no significant differences were observed in the mean Hb and mean Hct among the I-UCM, 60-s DCC, and 30-s DCC groups. Peak total serum bilirubin (TSB) levels were higher in the 60-s DCC group than in the I-UCM and PBCC groups (*p* = 0.017), but there was no difference between the 60-s DCC and 30-s DCC groups during the first week of life. The phototherapy requirement was higher in 60-s DCC than in IUCM and 30-sDCC (*p* = 0.001).

**Conclusions:**

Our findings demonstrated that PBCC, 30-s DCC, and I-UCM in neonates born by CS had no significant differences from each other on placental transfusion. The Hb and Hct in the neonates were higher after 60-s DCC than after PBCC.

## Introduction

After delivery, blood flow in the umbilical cord usually persists for a few minutes. Placental transfusion is the process of transferring blood from the placenta to the infant during the birthing process (1). The additional blood supply offered by intact cord circulation interacts with all organ systems to facilitate postdelivery adaptation and supports the complexities of internal stability throughout the transition (2). *In utero*, one-third of the fetus' blood volume is in the placenta at term gestation. The cardiac output to the lungs dramatically rises at birth, from 8 to 10% in fetal life to 50% in newborn life, necessitating a fast increase in blood volume to fill the capillary beds around each alveolus and help in lung tissue recruitment and expansion (3). The placenta serves as blood storage, designed to match the increased blood volume need (4). When the umbilical cord is clamped right after birth, a significant proportion of fetal blood stays in the placenta, resulting in a reduced red blood cell (RBC) count in the newborn. The two basic placental transfusion procedures used in the delivery room to enhance the RBC count in newborns are delayed cord clamping (DCC) and umbilical cord milking (UCM) (5). Physiologic-based cord clamping (PBCC) entails delayed umbilical cord clamping until the infant has started breathing or has received respiratory support, and the lung has been aerated (6). As a result, rather than waiting for a certain period, the PBCC concept refers to cord clamping only when breathing has been established (7). Placental transfusion is facilitated by spontaneous breathing and crying, which causes negative intrathoracic pressure and increases the gradient between the placental vasculature and the fetal right atrium (8). In vaginal deliveries, optimal placental transfusion can be achieved because of good uterine tone, making it feasible to wait at least 3 min after delivery to cut the cord (9, 10). Consonni et al. (11) reported that cord clamping after the first neonatal breath was correlated with high neonatal hematocrit (Hct) for cesarean sections (CSs) performed in labor but not for those performed electively. Strauss et al. (12) reported that the RBC count of CS-delivered newborns who received DCC after 60 s was lower than that of vaginally delivered infants. Furthermore, during DCC, inspiration enhances and prioritizes umbilical venous return from the placenta into the heart; thus, breathing might aid placental transfusion and may have been a problematic issue in earlier placental transfusion research (13). Nyberg et al. (14) discovered that during inspiration, umbilical blood flow was increased by 42% at the placental end of the umbilical cord. During a 60-s DCC, Katheria et al. (15) discovered no significant difference in Hct levels or short-term outcomes between preterm infants with and without assisted ventilation. Although it is accepted that spontaneous breathing contributes to the pressure gradient from the placenta to the neonatal circulation, its impact on placental transfusion is obscure (16–18). DCC is defined as delaying cord clamping until 30–180 s after delivery or until cord pulsing ceases to facilitate the transition from fetal to neonatal circulation (1, 19). The World Health Organization advises avoiding clamping the umbilical cord before one minute after birth, unless the newborn requires immediate resuscitation (20). For both vaginal and cesarean births, the American College of Obstetricians and Gynecologists advises at least 30–60 s of DCC (21). Furthermore, UCM efficiently accelerates placental transfusion at the moment of birth, perhaps delivering similar advantages as DCC (22). In light of this information, this study aimed to evaluate the impact of cord clamping after the first spontaneous breath on placental transfusion in neonates born by CS.

## Methods

Between August 2021 and February 2022, we enrolled all women who were admitted to the Acibadem Adana Hospital for CS with a live singleton pregnancy at ≥37.0 weeks of gestation. Informed consent was obtained from the parents of all infants. The Cukurova University School of Medicine gave its approval to the study protocol. Patients were assigned randomly. The interventions performed, such as PBCC, intact-UCM (I-UCM), 30-s DCC, and 60-s DCC, were assigned into four equal groups using computer-generated random numbers. Random numbers were noted on slips and put in opaque envelopes. To conceal their identity, the intervention group was sealed in opaque envelopes. The sealed envelope was opened by the nursing staff immediately before delivery. The intervention written the on slip was carried out by the obstetrics. Pregnancies complicated by fetal congenital anomalies, placenta previa, multiple pregnancies, and abruptio placenta, as well as suspected intrauterine growth restriction, and infants with hemolytic disease or requiring neonatal resuscitation were excluded. The primary outcomes were the mean neonatal hemoglobin (Hb), Hct, and total serum bilirubin (TSB) levels at 36 h after birth, as well as the requirement for phototherapy and peak TSB during the first week of life. Peak TSB was accepted as the highest level of the TSB and was sampled during the first week of life. The secondary outcomes were the respiratory rate (RR), heart rate (HR), and pulse oximetry oxygen saturation (SpO_2_) in the first 3 h after birth and required respiratory support [nasal continuous positive airway pressure (n CPAP) or high flow nasal cannula (HFNC)]. The complete blood count (CBC) was obtained using a Sysmex XT-1800i Analyzer (Sysmex Corporation, KOBE, Japan). The TSB levels were determined using a Siemens Dimension RxL Max Analyzer (Siemens Healthcare Diagnostics Inc., Newark, NJ, United States) *via* the diazo method. Cord blood gas analysis was performed for each infant after delivery using the Radiometer ABL800 Flex (Radiometer, Copenhagen, Denmark). The times to first urine and meconium were recorded. All maternal and neonatal data were obtained from medical records. A stopwatch timer was used to record the time to the first spontaneous newborn breath and all interventions in groups. In all interventions, the newborn was positioned on the maternal anterior thigh after cesarean delivery. PBCC was performed after spontaneous first neonatal breath and the time was recorded (6). The protocol for the I-UCM was as follows: the umbilical cord was held 25 cm from the umbilical stump and blood was pushed or “stripped” toward the newborn over a 2-s duration. Between each milking action, the cord was freed and allowed to refill with blood temporarily for a brief 2-s pause. This technique was repeated four times before the physician clamped the cord (23). The entire procedure was to be completed in no more than 20 s. Protocol for the DCC was as follows: the nursing staff recorded 30 or 60 s before asking the obstetrician to clamp the umbilical cord and the cord was clamped and cut after 30 or 60 s (30-s DCC and 60-s DCC, respectively) (21). Uterotonic medicines were given shortly after the cord was clamped to avoid postpartum hemorrhage. Maternal anemia was characterized as an Hb concentration of <11 g/dl before delivery (24). When the Hct surpassed the 95th percentile upper reference interval for gestational and postnatal age, neonatal polycythemia was considered. When Hb was less than the 5th percentile lower reference interval for gestational and postnatal age, neonatal anemia was diagnosed (25). The need for phototherapy was determined according to the hour-specific nomogram and associated American Academy of Pediatrics guidelines (26).

### Power analysis

The sample size was calculated by taking into account the reference values used in the previous studies on this subject (27). According to the power analysis with a 0.36 effect size, each group needed 29 newborns to show an Hb difference of at least 10% at the 0.05 α level and 90% power for a two-sided test. The sample size was raised to 32 newborns in each group due to the possibility of attrition.

## Statistical analysis

Continuous variables are summarized as the mean and standard deviation (SD), as well as the median and minimum-maximum when applicable, whereas categorical variables are given as numbers and percentages. The categorical variables were compared using the chi-square test and Fisher's Exact test. For continuous data, the Shapiro-Wilk test was employed to validate the normality of distribution. The Kruskal-Wallis test or one-way analysis of variance (ANOVA) was used to evaluate continuous variables between groups based on whether the statistical hypotheses were met. For normally distributed data, regarding the homogeneity of variances, the Tukey or Games-Howell test was used for multiple comparisons of groups. For non-normally distributed data, Bonferroni adjusted Mann-Whitney U test was used for multiple comparisons of groups. The IBM SPSS Statistics version 20.0 statistical software program was used for all analyses. Statistical significance was defined as a *p*-value of <0.05.

## Results

**Figure 1:**
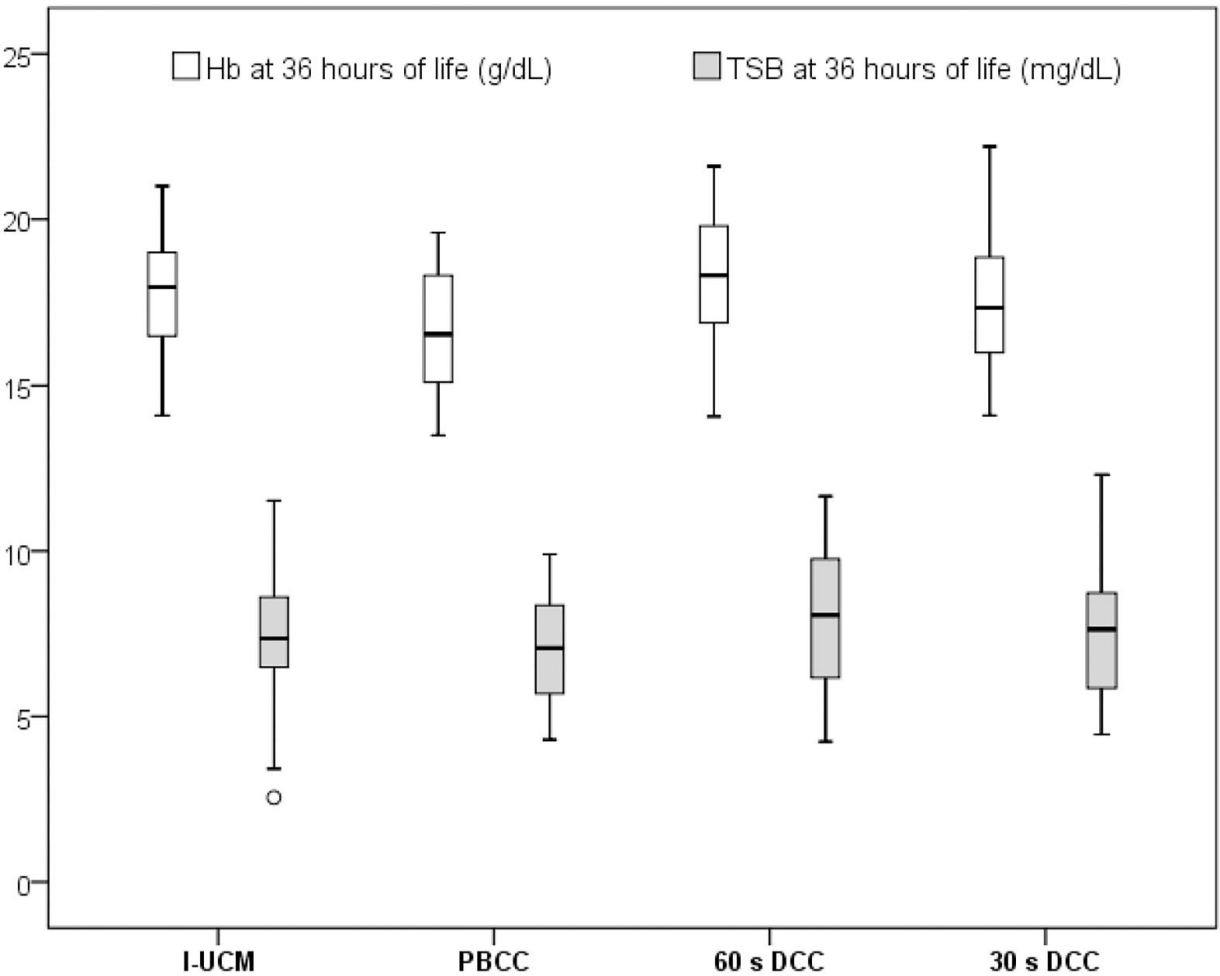
Hemaglobin (Hb) and total serum bilirubin (TSB) levels at 36 h of life 4.

During the study period, 128 infants were delivered by CS, of which 5 who did not meet the inclusion criteria were excluded. Among the 123 infants enrolled, 30, 31, 30, and 32 underwent I-UCM, PBCC, 60-s DCC, and 30-s DCC, respectively. The infants' demographic and laboratory data are presented in Table 1. No differences were noted in terms of the gestational age, gender, birth weight, and Apgar's scores at 1 and 5 min after delivery among the groups. Cord blood analysis revealed that lactate levels were significantly lower in the I-UCM group than in the 30-s DCC group (*p* = 0.049), but no other significant differences were observed between variables or groups. Maternal demographic and laboratory data are described in Table 2. No differences were observed in terms of maternal age, parity, gestational diabetes, or spontaneous onset of labor. However, gravida was significantly higher in the 60-s DCC group than in the PBCC group (*p* = 0.032). Maternal mean leukocytes, Hb, and Hct were similar between groups. The infants' postnatal laboratory data are presented in Table 3. Mean Hb and mean Hct were significantly higher in the 60-s DCC group than in the PBCC group (*p* = 0.028 and 0.019, respectively), but no differences were observed between I-UCM, 30-s DCC, and PBCC at 36 h of age. Further, no significant differences were seen between the mean Hb and mean Hct among the I-UCM, 60-s DCC, and 30-s DCC groups. No anemia was observed in any of the groups and the prevalence of neonatal polycythemia did not differ between groups. No significant differences were noticed in leukocyte, platelet, or TSB levels at 36 h of life among the groups. Although the peak TSB level was higher in the 60-s DCC group than in the I-UCM and PBCC groups (*p* = 0.017), no difference was seen between the 60-s DCC and 30-s DCC groups during the first week of life. The phototherapy requirement was higher in 60-s DCC than in I-UCM and 30-s DCC (*p* = 0.001). Mean Hb and mean TSB levels at 36 h of life are shown in Figure 1. The infants' clinical data for the first 3 h of life are presented in Table 4. The median onset time of spontaneous first breath of the neonates was similar between groups. No significant differences were noticed in SpO_2_, RR, HR, respiratory support, and the time to first urine and meconium among the groups.

**Table 1:** The infants' demographic and laboratory data.

	**I-UCM** ***N* = 30**	**PBCC** ***N* = 31**	**60-s DCC** ***N* = 30**	**30-s DCC** ***N* = 32**	***P***
Gestational age (weeks) (median, min-max)	38 (37–39)	38 (37–40)	38 (37–39)	38 (37–40)	0.415
Gender, male, *n* (%)	14 (47)	16 (52)	15 (50)	16 (50)	0.984
Birth weight (g, mean ± SD)	3317.8 ± 410.7	3279.3 ± 427.2	3396.9 ± 440.7	3426.2 ± 366.3	0.467
Apgar's score at 1 min (median, min–max)	9 (7–9)	9 (8–9)	9 (7–9)	9 (7–9)	0.515
Apgar's score at 5 min (median, min–max)	10 (9–10)	10 (9–10)	10 (9–10)	10 (8–10)	0.777
**Cord blood analysis**	
pH (mean ± SD)	7.37 ± 0.04	7.38 ± 0.11	7.37 ± 0.04	7.35 ± 0.05	0.422
PCO_2_ (mean ± SD)	39 ± 3.8	41.5 ± 5.8	39.8 ± 5.5	40.2 ± 6	0.376
PO_2_ (mean ± SD)	25.9 ± 7.2	24.6 ± 7.8	23.3 ± 6.9	26.9 ± 9.3	0.417
Base excess, mmol/L (median, min–max)	−2.3 (−5.1–2.2)	−1.4 (−7.2–0.6)	−2.4 (−4.6–0.9)	−2.5 (−8.3–1.8)	0.234
Bicarbonate, mmol/L (mean ± SD)	21.6 ± 1.3	21.9 ± 1.5	21.6 ± 0.9	21.1 ± 1.9	0.253
Lactate, mmol/L (mean ± SD)	1.3 ± 0.2	1.4 ± 0.3	1.5 ± 0.5	1.6 ± 0.7	**0.049** ^**a**^
Total bilirubin (mg/dL) (mean ± SD)	1.6 ± 0.5	1.6 ± 0.5	1.7 ± 0.6	1.7 ± 0.5	0.743
Hb (g/dL) (mean ± SD)	14.7 ± 1.4	14.9 ± 1.4	14.6 ± 1.1	15.2 ± 1.8	0.472
Hct (%) (mean ± SD)	45.5 ± 4.2	45.5 ± 4.1	44.6 ± 3.5	46.3 ± 5.3	0.650

I-UCM, intact-umbilical cord milking; PBCC, physiologic-based cord clamping; 60-s DCC, 60 s delay in cord clamping; 30-s DCC, 30 s delay in cord clamping; TB, Total bilirubin; Hb, hemoglobin; Hct, hematocrit; SD, standard deviation; pCO2, carbon dioxide partial pressure; pO_2_, oxygen partial pressure.

Kruskal Wallis (Gestational Age, APGAR Scores and Base Excess), Chi-Square Test (Gender) and Oneway ANOVA (others) were used.

^a^*p* < 0.05 for I-UCM vs 30-s DCC.

**Table 2:** Maternal demographic and laboratory data.

	**I-UCM** ***N* = 30**	**PBCC** ***N* = 31**	**60-s DCC** ***n* = 30**	**30-s DCC** ***n* = 32**	***P***
Maternal age (y, mean ± SD)	33.1 ± 3.2	32.4 ± 3.7	32.5 ± 3.5	33.1 ± 4.2	0.778
Gravida (median, min–max)	2 (1–7)	1 (1–4)	2 (1–5)	2 (1–4)	**0.032** ^**a**^
Parity (median, min–max)	2 (1–4)	1 (1–2)	2 (1–4)	2 (1–4)	0.057
Gestational diabetes, *n* (%)	2 (7)	1 (3)	4 (13)	5 (16)	0.476
Spontaneous onset of labor, *n* (%)	5 (17)	4 (13)	2 (7)	3 (9)	0.792
Leukocytes (x10^3^/μL) (mean ± SD)	9.6 ± 2.1	9.1 ± 1.5	9.8 ± 2	10 ± 2.2	0.356
Hb (g/dL) (mean ± SD)	12.3 ± 1.3	11.9 ± 1.1	12 ± 1.3	12.1 ± 1.1	0.633
Maternal anemia, *n* (%)	5 (17)	4 (23)	8 (27)	4 (12)	0.509
Hct (%) (mean ± SD)	36.9 ± 3.3	35.9 ± 3.1	36 ± 3.3	36.3 ± 2.9	0.601

I-UCM, intact-umbilical cord milking; PBCC, physiologic-based cord clamping; 60-s DCC, 60 s delay in cord clamping; 30-s DCC, 30 s delay in cord clamping; Hb, hemoglobin; Hct, hematocrit; SD, standard deviation.

Kruskal Wallis (Gravida and Parity), Chi-Square Test (Gestational diabetes, Spontaneous onset of labor, Maternal anemia) and Oneway ANOVA (others) were used.

^a^*p* < *0.05 for* PBCC vs 60-s DCC.

**Table 3:** The infants' postnatal laboratory data.

	**I-UCM** ***N* = 30**	**PBCC ** ***N* = 31**	**60-s DCC ** ***N* = 30**	**30-s DCC** ***N* = 32**	***P***
At 36 h of life	
TSB (mg/dL) (mean ± SD)	7.35 ± 2.06	7.05 ± 1.59	7.89 ± 2.02	7.65 ± 1.97	0.360
Hb (g/dL) (mean ± SD)	17.8 ± 1.8	16.7 ± 1.8	18.2 ± 2	17.6 ± 1.9	**0.028** ^**a**^
Hct (%)	50.8 ± 4.9	47.8 ± 5.5	51.9 ± 4.9	50.5 ± 5.5	**0.019** ^**a**^
Polycythemia (%)	0 (0)	0 (0)	0 (0)	1 (3)	0.999
Leukocytes (x10^3^/μL)	13.8 ± 3.9	14.5 ± 5	14.4 ± 4	15.2 ± 4.7	0.671
Platelets (x10^3^/μL)	281.7 ± 63.4	297.4 ± 61.2	292.4 ± 60.3	299.9 ± 78.2	0.718
**First week of life**	
Peak TSB (mg/dL) (mean ± SD)	11.2 ± 4	10.9 ± 3.6	13.7 ± 3.7	12.1 ± 3.3	**0.017** ^**a.b**^
Phototherapy (%)	0 (0)	3 (10)	7 (23)	0 (0)	**0.001** ^**b, c**^

I-UCM, intact-umbilical cord milking; PBCC, physiologic-based cord clamping; 60-s DCC,60 s delay in cord clamping; 30-s DCC, 30 s delay in cord clamping; TSB, total serum bilirubin; Hb, hemoglobin; Hct, hematocrit; SD, standard deviation.

Fisher's Exact Test (Polycythemia, Phototherapy) and Oneway ANOVA (others) were used.

^a^*p* < 0.05 for PBCC vs. 60-s DCC.

^b^*p* < 0.05 for I-UCM vs. 60-s DCC.

^c^*p* < 0.05 for 30-s DCC vs. 60-s DCC.

**Table 4:** Infants'clinical data.

	**I-UCM** ***N* = 30**	**PBCC** ***N* = 31**	**60-s DCC** ***N* = 30**	**30-s DCC** ***N* = 32**	***P***
Time to the first breath (sec) (median, min–max)	6 (2–18)	7 (2–27)	6 (2–24)	8 (2–25)	0.114
**Respiratory rate, beats/min**	
1st h	50.3 ± 6	50.3 ± 6.4	50.5 ± 6.5	51 ± 6.9	0.966
2nd h	49.8 ± 6.3	49.3 ± 7.6	48.9 ± 3.6	48.3 ± 5.7	0.810
3rd h	47.8 ± 4.5	48.3 ± 6	46.1 ± 2.3	47.7 ± 5.3	0.283
**Heart rate, beats/min**	
1st h	141.2 ± 13.5	138.8 ± 9.9	139.5 ± 10.5	141.6 ± 9.6	0.695
2nd h	138.7 ± 9.7	138.1 ± 9.7	137.3 ± 8.9	139.6 ± 7	0.774
3rd h	139.5 ± 8.1	135 ± 10.6	134.9 ± 7.2	135 ± 10.4	0.146
**SpO2**	
1st h	97.8 ± 1.6	98.1 ± 1.2	97.3 ± 2	97.7 ± 2.3	0.386
2nd h	98.5 ± 1.6	98.3 ± 1.7	98.1 ± 1.3	98.4 ± 1.1	0.777
3rd h	98.8 ± 1	98.4 ± 1.2	98.7 ± 1.2	98.6 ± 1	0.589
**Respiratory support**	
HFNC (%)	2 (7)	3 (10)	2 (7)	4 (12)	0.900
CPAP (%)	0 (0)	1 (3)	0 (0)	1 (3)	0.999
Time to first urine (h) (median, min–max)	5 (0–15)	6 (0–16)	5.5 (0–11)	6 (0–16)	0.690
Time to first meconium (h) (median, min–max)	8 (0–21)	8 (0–19.5)	9 (2–31)	11 (0–36)	0.974

I-UCM, intact-umbilical cord milking; PBCC, physiologic-based cord clamping; 60-s DCC, 60 s delay in cord clamping; 30-s DCC, 30 s delay in cord clamping; SpO2, pulse oximetry oxygen saturation; HFNC, high flow nasal cannula; nCPAP, nasal continuous positive airway pressure.

Kruskal Wallis (Time to the first breath, Time to first urine, Time to first meconium), Fisher's Exact Test (HFNC and CPAP) and Oneway ANOVA (others) were used.

## Discussion

This study evaluated the impact of cord clamping on placental transfusion and found that the mean Hb and mean Hct in CS-delivered neonates were significantly higher after 60-s DCC than after PBCC. However, the PBCC, 30-s DCC, and I-UCM had no significant differences from each other on placental transfusion. Interestingly, no significant differences were observed in the mean Hb and mean Hct among the I-UCM, 60-s DCC, and 30-s DCC groups.

In elective CS, umbilical cord clamping after 60 s has been found to be safe and useful for newborns (28). Hb and Hct levels in term neonates delivered by elective CS were shown to be higher after 60-s DCC in two earlier randomized controlled studies (29, 30). However, Zhou et al. showed that placental transfusion was decreased in CS in a systematic review and meta-analysis (31). DCC had no impact on placental-fetal transfusion in term CS, according to Kleinberg et al. (32). As a result, the impact of umbilical cord management on placental transfusion in CS has to be clarified. Neonatal breathing was a key determinant for placental transfusion during DCC, according to Boere et al. (33). Furthermore, Consonni et al. (11) found that PBCC was linked to a greater neonatal Hct after CS in labor, but not after elective CS in term neonates. The volume of placental transfusion toward the newborn varies significantly depending on whether ventilation happens before or after cord clamping because the amount of blood retained in the placenta is substantially larger in the latter (34). As a result, crying spontaneously before clamping the umbilical cord considerably increases the volume of placental transfusion (35). The pressure gradient from the placenta to the neonatal circulation was considered to be aided by negative intrathoracic pressure created by spontaneous respirations, in addition to gravity and uterine contractions. The impact of spontaneous breathing on placental transfusion, on the other hand, is still obscure (16–18). The term “physiological” cord clamping refers to a method of determining the length of a placental transfusion based on lung ventilation (36). In terms of placental transfusion, our findings showed that PBCC was comparable to 30-s DCC and I-UCM. The increase in pulmonary blood flow may shift a significant volume of blood from systemic blood flow through fetal shunts during lung inflation; thus, if lung aeration occurs while the cord is intact, then a significant volume of cord blood might be added to the infant to compensate for the shifted volume (37). It is still up for discussion whether placental-to-neonatal transfusion produces net increases in neonatal blood volumes with DCC during a cesarean birth. The lack of uterine contractions before birth may affect the efficiency of placental-to-neonatal transfusion in elective CS (38). Due to strong uterine tone, optimal placental transfusion is possible in vaginal births, making it possible to wait for least 3 min after delivery before cutting the cord (9, 10). Even if DCC is feasible, because the uterus is cut open in CS, the newborn may not get enough placental transfusion as compared to vaginal birth (22). In our study, although the mean Hb and mean Hct following 60-s DCC were increased, this increase did not reach statistical significance when compared with that of 30-s DCC and I-UCM. This may be explained by the lack of uterine contractions in CS, which may influence transfusion performance. In addition, all infants in our study breathed during the procedure, and the mean onset times to their first breath were similar among groups. Our results showed that neonates' spontaneous first breath may be more important for placental transfusion than clamping time for neonates born by CS. If umbilical cord clamping is to be postponed, there is now compelling evidence that the time of umbilical cord clamping should be determined by the infant's physiology (particularly breathing) than by a stopwatch (39). When compared to DCC, Katheria et al. (23) found that UCM during CS enhanced blood flow and organ perfusion by providing more placental transfusion, as evaluated by improved superior vena cava flow and higher admission Hb in preterm neonates. Another research comparing UCM with DCC for preterm newborns found that neonatal Hb and Hct levels were similar. UCM and DCC are two ways of placental transfusion that are similar (40). UCM effectively accelerates placental transfusion at the moment of birth, with advantages comparable to DCC (22). Because the uterus is not contracting forcefully during cesarean births, UCM may be a more effective method of blood transfer (23). “UCM” regardless of whether cord milking occurs while the umbilical cord is still attached to the placenta (I-UCM) or after it has been cut and separated from the placenta (C-UCM) (8). In our study, I-UCM and DCC for term neonates resulted in similar Hb and Hct outcomes, but further controlled trials are required for a full assessment. In our previous study, when comparing I-UCM to C-UCM with immediate cord clamping in neonates delivered >35 weeks' gestation, I-UCM was found to be the better option (41).

Overtransfusion, polycythemia, hypervolemia, and hyperbilirubinemia are all theoretical risks of placental transfusion from either DCC or UCM (42, 43). Yang et al. (44) demonstrated that the mean peak neonatal transcutaneous bilirubin levels were significantly higher among term neonates who underwent DCC. However, no differences were noted in mean peak TSB levels or in the need for phototherapy between groups. In our study, no significant variations in mean TSB levels at 36 h of life were found between the groups. While the 60-s DCC group had higher peak TSB levels than the I-UCM and PBCC groups, there was no difference between the 60-s DCC and 30-s DCC groups throughout the first week of life. The 60-s DCC group required more phototherapy than the I-UCM and 30-s DCC groups. There was also no increase in the incidence of polycythemia.

Waiting until the infant has taken a breath before clamping the cord improves clinical outcomes (45). Cord clamping time affects both the SpO_2_ and HR of newborns during the initial transition (46). In a recent study, researchers evaluated immediate and DCC in term infants delivered by elective CS and discovered that DCC had no effect on SpO_2_ or HR (47). Cavallin et al. (29) found that preductal oxygen saturation and HR during the first 10 min of life in neonates born at 39 weeks' gestation delivered by elective CS were not significantly different between DCC and early cord clamping (ECC). Likewise, in the first 3 h following birth, we detected no differences in SpO_2_ levels or HR from hour to hour. The effects of DCC on umbilical cord blood gas levels during cesarean births are little known. Both in vaginal and cesarean births, arterial cord blood pH, bicarbonate, and base excess were dropped considerably after 3-min DCC when compared to ECC values in a cohort of term infants born vaginally or by elective cesarean. When comparing cesarean births to vaginal deliveries, DCC was linked to an increase in pCO_2_ and lactate (48). Nudelman et al. (49) conducted a comprehensive evaluation of five trials in which blood gases were collected up to 120 s after DCC in term newborns. The cord blood gas values were either unaffected or had a modest and clinically negligible influence as a result of the delay. We found no significant difference in the umbilical cord blood gas values of pH, bicarbonate, pCO_2_, pO_2_, and base excess between the groups. Lactate was significantly higher in the 30-s DCC group when compared with that in the I-UCM group, but it did not differ between the other groups. DCC supports the fetal-to-neonatal transition by keeping oxygen supply and left ventricular preload until the lungs are aerated (39). When compared to DCC, I-UCM enhances pulmonary blood flow soon after delivery, aiding with lung expansion at the commencement of respiration and allowing for an earlier onset of breathing (8). However, there was no difference in the postdelivery respiratory support (HFNC and n-CPAP) between the groups in our study. Further, all infants in our study breathed during the procedure, and the mean onset times to their first breath were similar among groups.

The strength of this study is that, to our knowledge, it is the first to simultaneously evaluate different methods of umbilical cord clamping, i.e., PBCC vs. DCC vs. I-UCM in neonates born by elective CS. We acknowledge that our study is limited by a relatively small sample size. Controlling for confounders in a study involving multiple comparisons is exceptionally difficult. Although most of the infants included in the study were born by elective CS, some of them were born by CS in labor. This may be considered another limitation of the study, as it may complicate the interpretation of the results. In addition, we could evaluate only Hb and Hct levels as indicators of placental transfusion and its short-term outcomes.

## Conclusion

Our findings showed that PBCC, 30-s DCC, and I-UCM in neonates born by CS had no significant differences from each other on placental transfusion. Hb and Hct in the neonates were higher after 60-s DCC than after PBCC. Strikingly, Hb and Hct in 60-s DCC were not significantly higher than those in 30-s DCC and I-UCM. Further studies are required to determine the optimal time for cord clamping after the first spontaneous breath in term infants, as well as its potential clinical impact and long-term outcomes.

## Data availability statement

The original contributions presented in the study are included in the article/supplementary material, further inquiries can be directed to the corresponding author/s.

## Ethics statement

The studies involving human participants were reviewed and approved by Cukurova University School of Medicine. Written informed consent to participate in this study was provided by the participants' legal guardian/next of kin.

## Author contributions

The hypothesis of the study belongs to HK. It was also designed and written the study by HK. DP and MS collected the information of infants and mothers and followed cord management during CS. SCD, as an obstetrician, managed the work in harmony with the obstetrics department. All authors contributed to the article and approved the submitted version.

## Conflict of interest

The authors declare that the research was conducted in the absence of any commercial or financial relationships that could be construed as a potential conflict of interest.

## Publisher's note

All claims expressed in this article are solely those of the authors and do not necessarily represent those of their affiliated organizations, or those of the publisher, the editors and the reviewers. Any product that may be evaluated in this article, or claim that may be made by its manufacturer, is not guaranteed or endorsed by the publisher.
